# Supplementary material for: Selective light absorber-assisted single nickel atom catalysts for ambient sunlight-driven CO2 methanation
Source: Nat Commun. 2019 May 29;10:2359. doi: 10.1038/s41467-019-10304-y (PMC6541650; doi:10.1038/s41467-019-10304-y)
Supplement: Supplementary file 1 — Supplementary Information [file 41467_2019_10304_MOESM1_ESM.pdf]

Selective light absorber-assisted single nickel atom catalysts for ambient sunlight-driven CO_2 methanation

Yaguang Li et al.

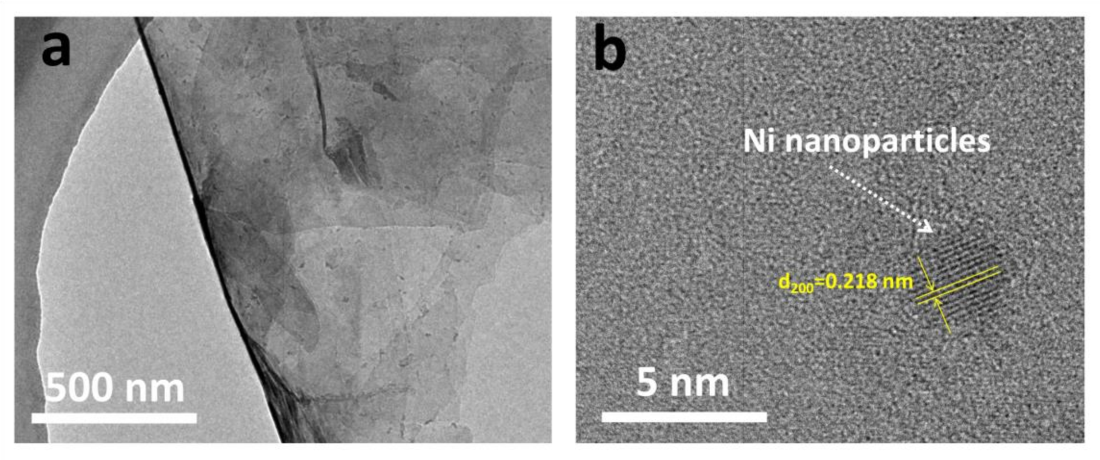

Supplementary Figure 1. Characterization of Ni/Y2O3 nanosheets. **a, b** TEM and HRTEM images of Ni/Y_2O_3 nanosheets.

The TEM image of Ni/Y_2O_3 nanosheets showed some nanoparticles distributed on the nanosheets (Supplementary Figure 1a) and the HRTEM image revealed the crystalline nature of the nanoparticles with 0.218 nm of lattice spacing, cresponding to the (002) plane of metallic Ni,[1] thus, confirming that the ctystalline precipitations on nanosheets are Ni nanoparticles.

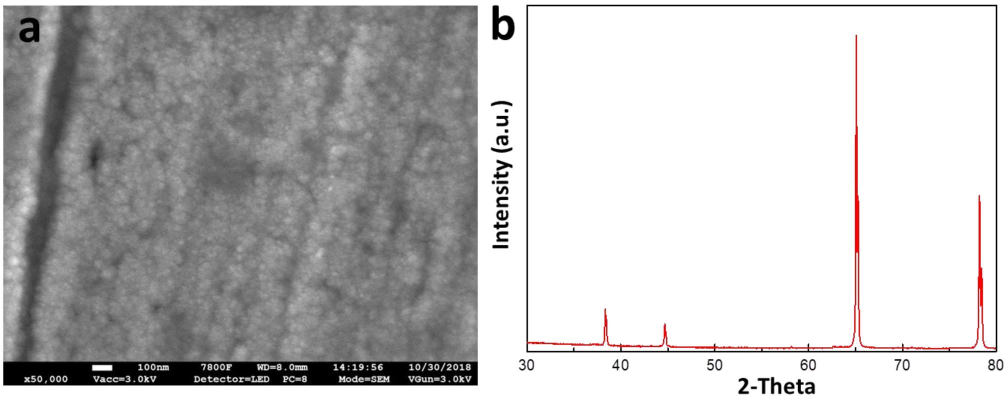

Supplementary Figure 2. Characterization of selective light absorber. **a** SEM image and **b** XRD pattern of selective light absorber.

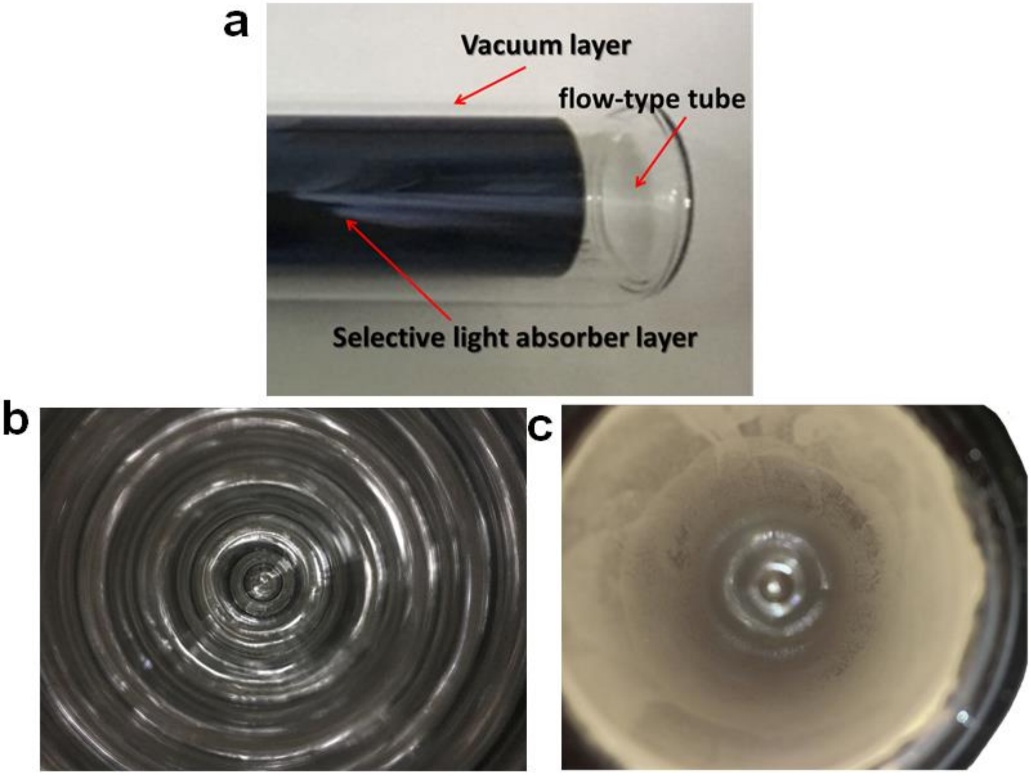

Supplementary Figure 3. Characterization of selective light absorber coated quartz tube. **a** Photograph of selective light absorber layer coated on quartz tube. **b, c** Photograph of selective light absorber layer assisted quartz tubes coated without and with catalysts.

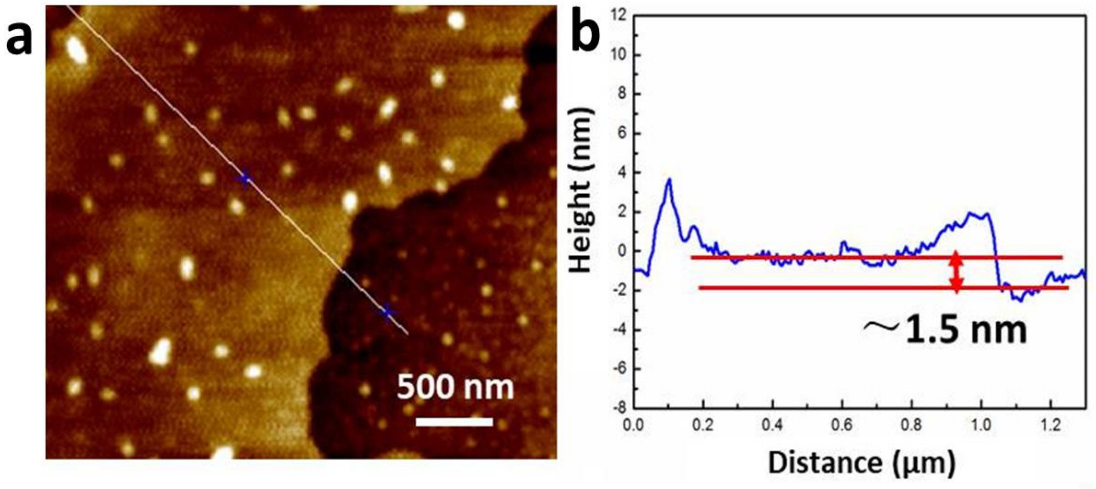

Supplementary Figure 4. Characterization of SA Ni/Y_2O_3 nanosheets. **a** AFM image, **b** height profile of SA Ni/Y_2O_3 nanosheets.

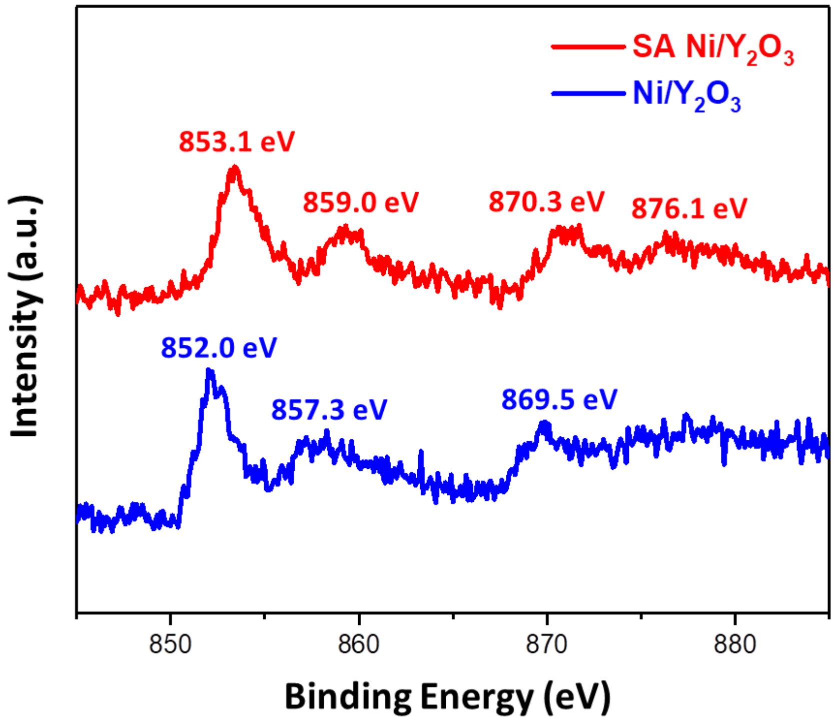

Supplementary Figure 5. The Ni 2p XPS spectra of SA Ni/Y_2O_3 nanosheets (red) and Ni/Y_2O_3 nanosheets (blue).

The binding energy of Ni $2p_{3/2}$ is 853.1 eV for SA Ni/Y_2O_3 nanosheets, a little lower than that of NiO (853.7 eV),[1] revealing the oxidation state of Ni in SA Ni/Y_2O_3 nanosheets. The binding energy of Ni $2p_{3/2}$ is 852.0 eV for Ni/Y_2O_3 nanosheets, similar to the metallic Ni (852.0 eV),[1] confirming the metallic state of Ni in Ni/Y_2O_3 nanosheets.

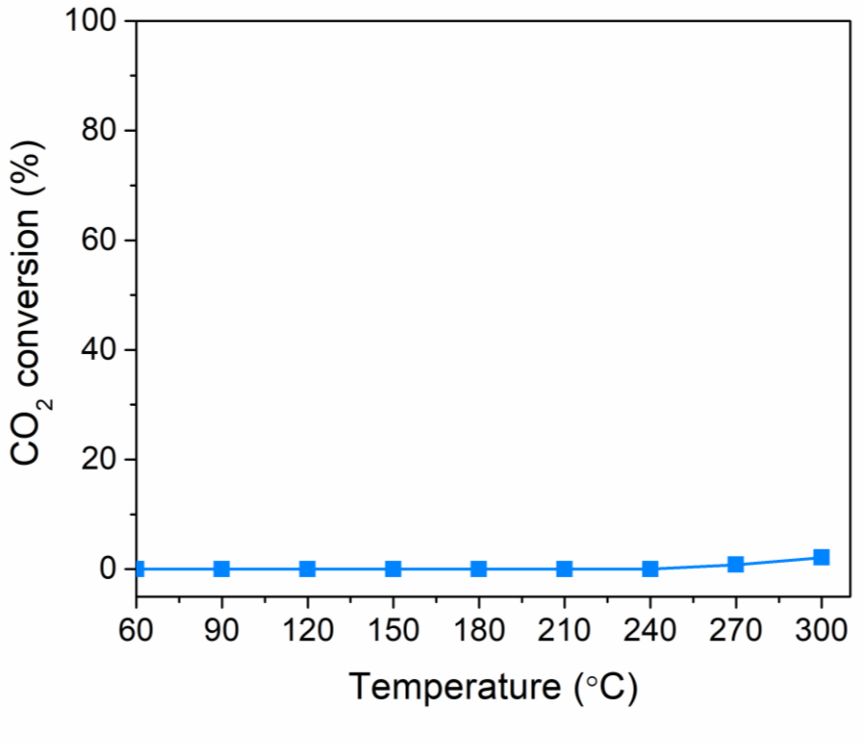

Supplementary Figure 6. Thermal CO_2 conversion of Y_2O_3 nanosheets. Reaction condition: 100 mL min^{-1} of reaction gas (2.5% CO_2 +10 % H_2 + 87.5% N_2), 100 mg of catalysts.

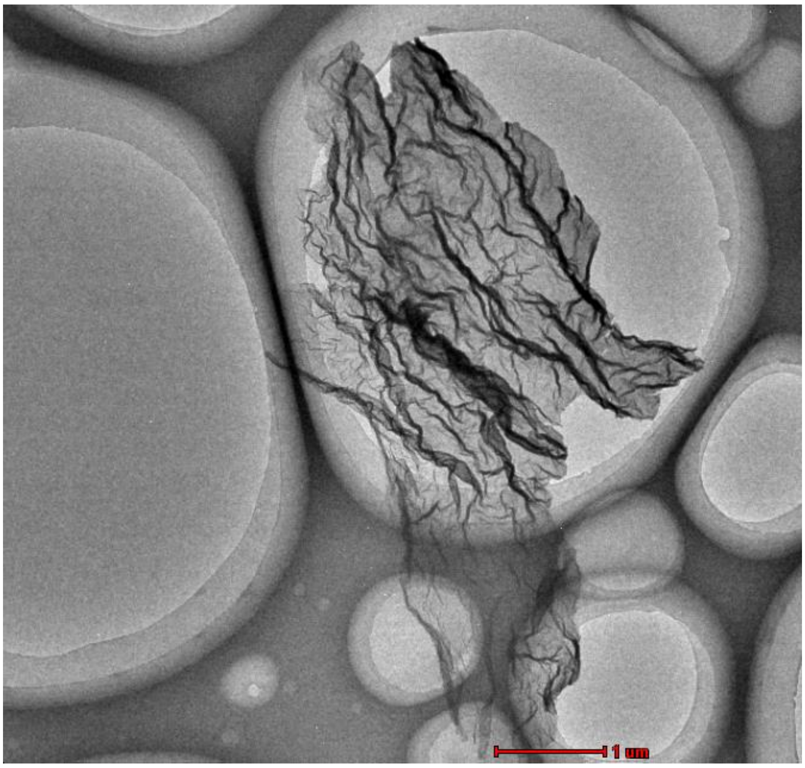

Supplementary Figure 7. TEM image of SA Ni/Y_2O_3 nanosheets after 90 hours' heating/cooling thermal CO_2 methanation test.

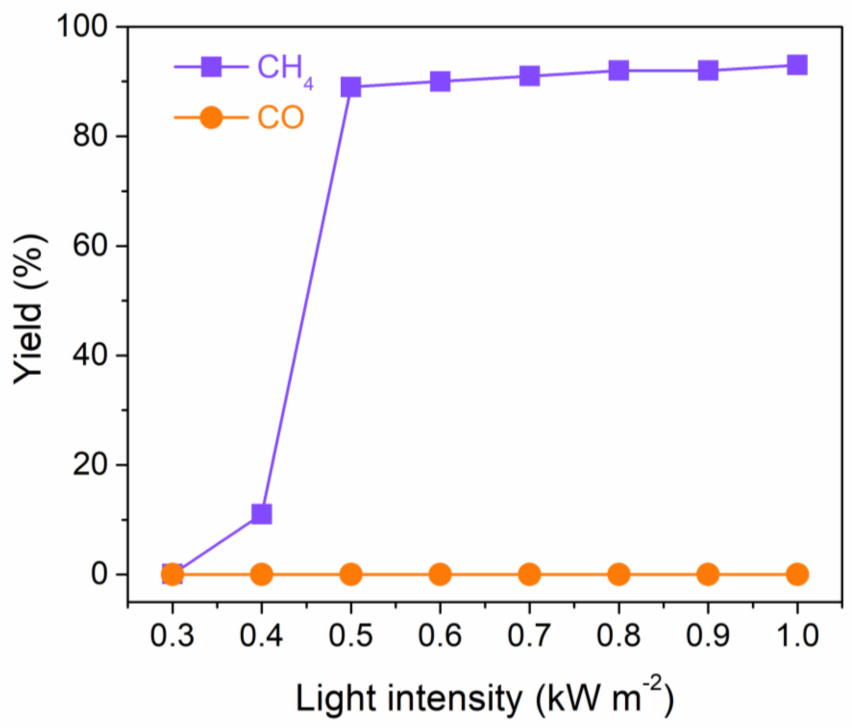

Supplementary Figure 8. CH_4 and CO yields in photothermal CO_2 methanation under different densities of solar light irradiation.

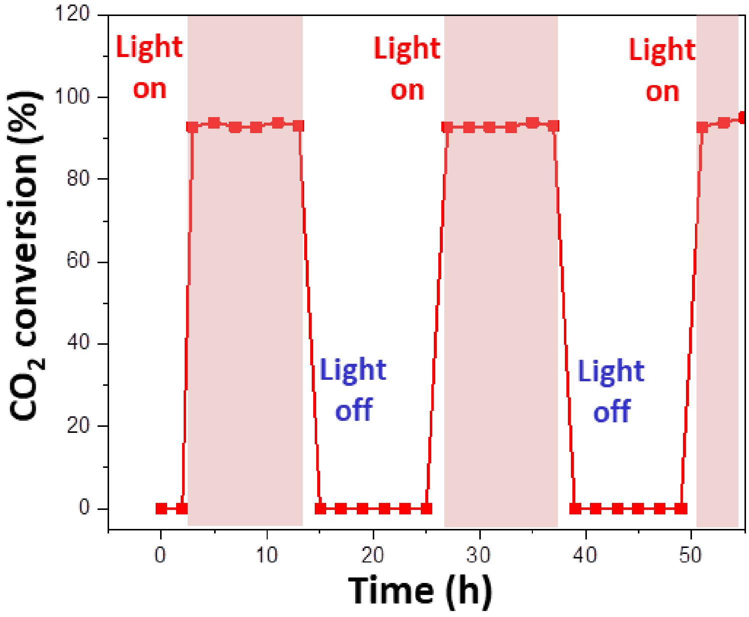

Supplementary Figure 9. CO_2 hydrogenation performance versus reaction time, under 1 kW m^{-2} of simulated solar light irradiation.

Supplementary Table 1. The sunlight-driven temperature of different materials in air and in vacuum respectively. The sunlight intensity is 1.0 kW m^{-2}.

Materials	CNT	Graphene	2wt% Au/Al_2O_3	4wt% Ni/Y_2O_3	CuO	2wt% Ru/Al_2O_3	Selective light absorber
Temperature in air (℃)	70	79	70	78	82	64	260
Temperature in vacuum (℃)	98	86	88	97	103	82	300

Supplementary Table 2. Physicochemical properties of the as-prepared samples.

Catalysts	Metal loading (wt%)[a]	S_{BET} (m^2 g^{-1})[b]
3.9 wt% SA Ni/Y_2O_3	3.9	425
Ni/Y_2O_3	4.0	409

a, analyzed by ICP-AES.

b, obtained by N_2 adsorption-desorption method through Brunauer-Emmett-Teller (BET) equation.

Supplementary Table 3. The thermal CO_2 methanation performances of different catalysts.

Catalysts	Temperature (℃)	TOF (CO_2)	References
SA Ni/Y_2O_3	200	0.023	This work
Ni/Co_3O_4	200	0.003	[1]
Co NP	200	0.005	[2]
Ni/Al_2O_3	200	0.006	[3]
Ni/VO_x	210	0.0023	[4]
Ni/ZrO_2	235	0.058	[5]
Ni/Al_2O_3	300	5.7	[6]
Ni/SiO_2	300	1.61	[7]
Co/ZrO_2	400	0.2	[8]
NiFe	300	5.9	[9]

References

1 Wang, Y., Arandiyan, H., Scott, J., Dai, H. & Amal, R. Hierarchically Porous Network-Like Ni/Co_3O_4: Noble Metal-Free Catalysts for Carbon Dioxide Methanation. *Advanced Sustainable Systems* **2**, 1700119 (2018).

2 Beaumont, S. K. *et al.* Combining in Situ NEXAFS Spectroscopy and CO_2 Methanation Kinetics To Study Pt and Co Nanoparticle Catalysts Reveals Key Insights into the Role of Platinum in Promoted Cobalt Catalysis. *J. Am. Chem. Soc.* **136**, 9898-9901 (2014).

3 Meng, X. *et al.* Photothermal conversion of CO_2 into CH_4 with H_2 over Group VIII nanocatalysts: an alternative approach for solar fuel production. *Angew. Chem. Int. Ed.* **53**, 11478-11482 (2014).

4 Lu, X. *et al.* VOx promoted Ni catalysts supported on the modified bentonite for CO and CO_2 methanation. *Fuel. Process. Technol.* **135**, 34-46 (2015).

5 Jia, X., Zhang, X., Rui, N., Hu, X. & Liu, C.-j. Structural effect of Ni/ZrO_2 catalyst on CO_2 methanation with enhanced activity. *Appl. Catal. B-Environ.* **244**, 159-169 (2019).

6 He, L., Lin, Q., Liu, Y. & Huang, Y. Unique catalysis of Ni-Al hydrotalcite derived catalyst in CO_2 methanation: cooperative effect between Ni nanoparticles and a basic support. *J. Energy. Chem.* **23**, 587-592 (2014).

7 Aziz, M. A. A. *et al.* Highly active Ni-promoted mesostructured silica nanoparticles for CO_2 methanation. *Appl. Catal. B-Environ.* **147**, 359-368 (2014).

8 Li, W. *et al.* ZrO_2 support imparts superior activity and stability of Co catalysts for CO2 methanation. *Appl. Catal. B-Environ.* **220**, 397-408 (2018).

9 Pandey, D. & Deo, G. Effect of support on the catalytic activity of supported Ni–Fe catalysts for the CO_2 methanation reaction. *J. Ind. Eng. Chem.* **33**, 99-107 (2016).
